# Associated factors of stress, urgency, and mixed urinary incontinence in a geriatric outpatient assessment of older women with hip fracture

**DOI:** 10.1007/s41999-024-00997-w

**Published:** 2024-05-27

**Authors:** Aino T. Hellman-Bronstein, Tiina H. Luukkaala, Seija S. Ala-Nissilä, Maria S. Nuotio

**Affiliations:** 1 Department of Geriatric Medicine, Turku University Hospital and University of Turku, Turku, Finland; 2 Research, Development and Innovation Center, Tampere University Hospital, Tampere, Finland; 3 Health Sciences, Faculty of Social Sciences, Tampere University, Tampere, Finland; 4 Department of Obstetrics and Gynecology, Turku University Hospital and University of Turku, Turku, Finland; 5 Department of Geriatric Medicine, Seinäjoki Central Hospital, Seinäjoki, Finland

**Keywords:** Stress urinary incontinence, Urgency urinary incontinence, Mixed urinary incontinence, Urinary incontinence, Hip fracture

## Abstract

**Purpose:**

Urinary incontinence (UI) is known to be common among older female hip fracture patients. Little is known about different subtypes of UI among these patients. Our aim was to identify factors associated with subtypes of UI in a cross-sectional design.

**Methods:**

1,675 female patients aged ≥ 65 and treated for their first hip fracture in Seinäjoki Central Hospital, Finland, during 2007–2019, were included in a prospective cohort study. Of these, 1,106 underwent comprehensive geriatric assessment (CGA), including questions on continence, at our geriatric outpatient clinic 6 month post-fracture. A multivariable-adjusted multinomial logistic regression model was used to examine factors associated with UI subtypes.

**Results:**

Of the 779 patients included, 360 (46%) were continent and 419 (54%) had UI 6-month post-fracture. Of the women with UI, 117 (28%) had stress UI, 183 (44%) had urgency UI and 119 (28%) had mixed UI, respectively. Mean age of the patients was 82 ± 6,91. In multivariable analysis, depressive mood and poor mobility and functional ability were independently associated with stress UI. Fecal incontinence (FI) and Body Mass Index (BMI) over 28 were independently associated with urgency UI. Mixed UI shared the aforementioned factors with stress and urgency UI and was independently associated with constipation.

**Conclusions:**

Mixed UI was associated with most factors, of which depressive mood and impaired mobility and poor functional ability were shared with stress UI, and FI and higher BMI with urgency UI. CGA is key in assessing UI in older hip fracture patients, regardless of subtype.

## Key Summary Points

**Aim:**

The aim was to examine factors associated with different subtypes (stress, urgency and mixed) of urinary incontinence among older female hip fracture patients using a cross-sectional design.

**Findings:**

Urinary incontinence was frequent, and the most common type was urgency incontinence. Stress incontinence and urgency incontinence were associated with different modifiable risk factors, all of which were associated also with mixed incontinence.

**Message:**

Different risk factors converge in mixed urinary incontinence. Comprehensive geriatric assessment is key in the assessment of UI in older hip fracture patients.

## Introduction

Urinary incontinence (UI), defined as any reported involuntary loss of urine according to the International Continence Society [1], affects women disproportionately compared to men, and its prevalence increases with advancing age. Nearly 40% of women older than 60 years and 80% of women residing in long-term care have UI [2, 3]. UI is one of the most common geriatric syndromes and associated with adverse outcomes, such as decline in quality of life, disability, and depression. As opposed to UI as an urogynecologic condition in younger women, factors leading to the development of UI in older women are complex and often related to multifactorial health conditions and functional limitations. Despite high prevalence, UI remains under-reported and -managed. [2, 4, 5]

UI can be further classified into stress urinary incontinence (SUI), urgency urinary incontinence (UUI), and mixed urinary incontinence (MUI). SUI is defined as complaint of involuntary loss of urine on effort or physical exertion such as coughing or lifting, UUI as complaint of involuntary loss of urine associated with urgency, a sudden desire to void, and MUI as combination of the other two [1]. SUI is known to be the most prevalent subtype among women over-all reaching its peak in the fifth decade [6]. In Nurse’s Health study with over 10,000 participants affected by UI 56% of women aged between 41 and 83 years reported SUI, while 23% reported UUI and 21% MUI, respectively [7]. However, the prevalences of UUI and MUI exceed that of SUI in older age groups [6, 8]. There is evidence that MUI might have a greater physical and mental health burden compared to UUI or SUI in isolation [7, 9–11]. Of note, urgency with or without UI often combined with increased daytime urinary frequency and nocturia, is regarded as the key symptom of the overactive bladder (OAB) syndrome [12].

UI, and especially UUI, has been recognized as a risk factor for falls in older individuals [13, 14]. Hip fracture, one of the most devastating consequences of a fall, is a common and severe injury among older women with well-known consequences of increased mortality, morbidity, and health care costs [15, 16]. We have previously demonstrated over-all UI to be highly prevalent among older female hip fracture patients, and associated with older age, cognitive disorder, functional disability, depressive mood, and constipation [17]. Literature on associated factors of different UI-subtypes remains scarce, especially in this patient population. The treatment modalities available for UI depend on the subtype. However, since the relationship between the UI subtypes is complex, especially among older patients [6, 18], our aim was to examine factors associated with different subtypes of UI, and how they might differ between the groups in this vulnerable patient population.

## Methods

### Study population

This study was a retrospective analysis of prospectively collected data. As described in our previous works [17, 19], the study population consisted of 1,675 older women suffering their first hip fracture between September 2007 and January 2019. Patients were aged ≥ 65 and managed in Seinäjoki Central Hospital, which is the only hospital providing acute surgical care in the Hospital District of Southern Ostrobothnia, with a catchment area of approximately 200,000 residents. Pathologic and periprosthetic fractures were excluded from the study. All the patients were invited to a comprehensive geriatric assessment (CGA) at our out-patient clinic, which took place in a median time of 6 months (IQR 4–6 months) after the fracture. All participants or their representatives gave informed consent. The study design was approved by the Ethics Committee of the Hospital District of Southern Ostrobothnia. The study complies with the ethical standards of the Declaration of Helsinki and its later amendments.

**Fig. 1:**
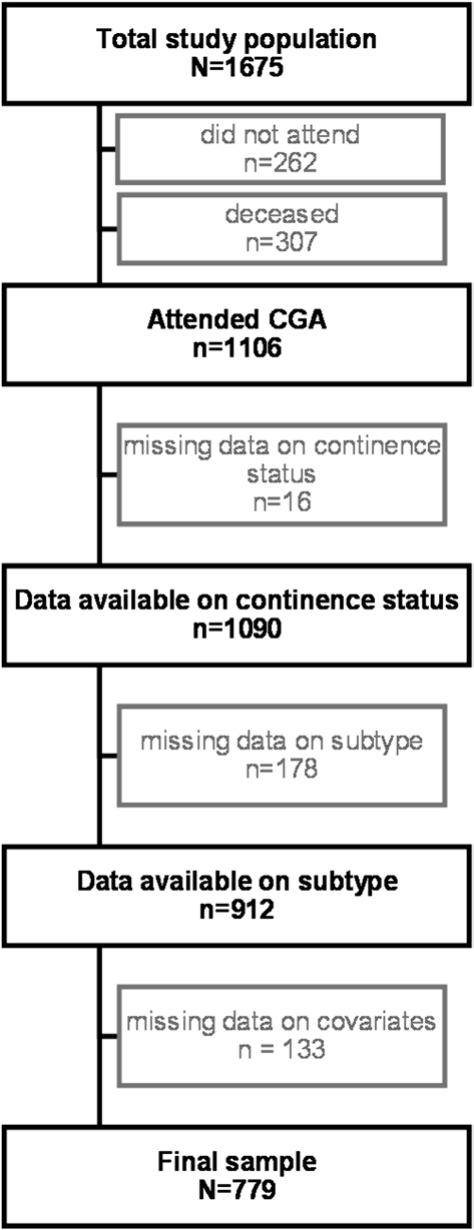
Flow chart of the study population

A total of 1,106 women attended the CGA. Data on continence status or subtype of UI was missing from 194 patients, leaving 912 women with the data on continence at 6 month post-fracture. After further exclusions of patients with missing data on covariates, a final sample of 779 women was generated for the statistical analyses of the associations of outpatient domains with the different UI subtypes. The study population is presented in detail in Fig. 1.

### Study protocol and variables

The study protocol and the variables used in our study have been described in greater detail elsewhere [17, 20]. Briefly, a trained geriatric nurse interviewed the patients or their representatives during hospitalization and retrieved data from the medical records. The preoperative American Society of Anesthesiologists (ASA) risk scores were used to assess general health at the time of the fracture. There are five classes: (1) healthy person, (2) mild systemic disease, (3) severe systemic disease, (4) severe systemic disease that is a constant threat of life, and (5) a moribund person who is not expected to survive without surgery [21]. ASA scores were used as an indicator of comorbidity in our analysis.

At the outpatient clinic, CGA was performed by a multidisciplinary team consisting of a geriatric nurse, a physiotherapist and a geriatrician or a trainee in geriatric medicine. Both the patients and their next of kin or carer were invited to the clinic [20]. The geriatric nurse carried out the interview and the assessment of the domains with the selected tools. We used official Finnish translations of well-known and validated tools in the CGA. Cognitive function was measured with the Mini Mental State Examination (MMSE) [22], nutritional status was assessed using the Mini Nutritional Assessment Short Form (MNA-SF) [23], depressive mood with the 15-item Geriatric Depression Scale (GDS-15) [24], and Instrumental Activities of Daily Living (IADL) by Lawton and Brody [25]. Patient’s body mass index (BMI) was also measured during the assessment and the number of regularly taken medications elicited. The physiotherapist’s assessment of the patient’s physical performance included the Timed up and go-test (TUG) assessing mobility, balance, and risk of falls [26], the Elderly Mobility Scale (EMS) which represents patient’s mobility and ability to perform basic activities of daily living [27], and grip strength as a measure of muscle strength. Grip strength was measured in the stronger hand with a Jamar dynamometer and defined as weakened if less than 16 kg [28]. In cases of MNA-SF and TUG, variables were dichotomized for statistical purposes.

UI was defined as any reported involuntary loss of urine, and FI as any reported involuntary loss of faeces, as in our previous works [17, 19]. Questions based on different UI subtypes were used to determine the likely UI type the patient was affected by. SUI was defined as having urinary leakage during physical exertion such as lifting or coughing, UUI as having urinary leakage associated with urgency symptom i.e. a sudden urge to void, and MUI as a combination of the other two [29]. Data on self-reported constipation (yes or no) and new falls after fracture (yes or no) were also elicited. Considering a substantial proportion of respondents were old and/or cognitively impaired, simple survey-like questions were preferred to using structured questionnaires. The categorization of outpatient domains is presented in Tables 1 and 2.

**Table 1:** Distribution of the outpatient domains according to continence status (*n* = 779) after 6 month follow-up. Differences between continence groups (*p* value) were tested using Pearson Chi-square test or Fisher’s exact test

Predictor	Continent		Stress incontinent		Urgency incontinent		Mixed incontinent		*p*
	*n* = 360		*n* = 117		*n* = 183		*n* = 119		
	*n*	(%)	*n*	(%)	*n*	(%)	*n*	(%)	
Age									0.016
65–79	139	39	38	33	47	26	34	29	
80–89	186	52	67	57	109	60	63	53	
≥ 90	35	10	12	10	27	15	22	19	
ASA									0.013
1–2	83	23	12	10	37	20	15	13	
3–5	272	76	101	87	144	79	103	87	
Regular medication									< 0.001
< 4	46	13	8	7	14	8	4	3	
4–10	233	65	68	58	101	55	61	51	
> 10	81	23	41	35	68	37	54	45	
MMSE									0.002
Normal (24–30)	172	48	50	43	79	43	33	28	
Abnormal (< 24)	188	52	67	57	104	57	86	72	
IADL									< 0.001
No difficulties (8)	104	29	19	16	28	15	13	11	
Difficulties (0–7)	256	71	98	84	155	85	106	89	
GDS-15									< 0.001
Normal (0–6)	323	90	81	69	147	80	86	72	
Depressed (> 6)	37	10	36	31	36	20	33	28	
EMS									< 0.001
Normal (14–20)	318	88	80	68	142	78	68	67	
Abnormal (< 14)	42	12	37	32	41	22	51	43	
TUG									< 0.001
Normal (1–2)	157	44	33	28	70	38	36	30	
Abnormal (3–5)	192	53	78	67	102	56	69	58	
Not known	11	3	6	5	11	6	14	12	
Grip strength, stronger hand									< 0.001
Normal (≥ 16 kg)	96	27	28	24	47	26	24	20	
Abnormal (< 16 kg)	161	45	56	48	98	54	82	69	
Not known	103	29	33	28	38	21	13	11	
MNA-SF									< 0.001
Normal (12–14)	191	53	55	47	96	53	35	29	
Poor nutrition (< 12)	169	47	62	53	87	48	84	71	
Fecal incontinence									< 0.001
No	346	96	105	90	163	89	98	82	
Yes	14	4	12	10	20	11	21	18	
Constipation									< 0.001
No	151	42	45	39	61	33	38	32	
Yes	104	29	39	33	79	43	70	59	
Not known	105	29	33	28	43	24	11	9	
BMI									< 0.001
23–28	167	46	55	47	79	43	41	35	
< 23	110	31	22	19	37	20	28	24	
> 28	83	23	40	34	67	37	50	42	
New falls after fracture									0.012
No	280	78	81	69	121	66	80	67	
Yes	80	22	36	31	62	34	39	33	

*ASA* American Society of Anesthesiologists-risk score, *MMSE* Mini Mental State Examination, *IADL* Instrumental Activities of Daily Living, *GDS-15* Geriatric depression scale, *EMS* Elderly Mobility Scale, *TUG* Timed Up and Go-test, *MNA-SF* Mini Nutritional Assessment Short Form, *BMI* Body Mass Index. Small numbers of missing values (*n* < 15) are not shown in table

**Table 2:** Multivariable-adjusted associations of outpatient domains with different types of UI 6 month post fracture among female hip fracture patients (*n* = 779). Reference group in multinomial logistic regression analysis was no incontinence (*n* = 360)

Predictor	Stress incontinent (*n* = 117)				Urgency incontinent (*n* = 183)				Mixed incontinent (*n* = 119)			
	*n*	(%)	OR	(95% CI)	*n*	(%)	OR	(95% CI)	*n*	(%)	OR	(95% CI)
Age												
65–79	38	33	1		47	26	1		34	29	1	
80–89	67	57	1	(0.59–1.71)	109	60	1.59	(1.00–2.54)	63	53	0.85	(0.48–1.50)
≥ 90	12	10	0.77	(0.32–1.84)	27	15	2	(0.99–4.06)	22	19	1.01	(0.45–2.27)
ASA score												
1–2	12	10	1		37	20	1		15	13	1	
3–5	101	87	2.04	1.00–4.19	144	79	0.87	0.52–1.45)	103	87	1.33	(0.66–2.67)
Regular medication												
< 4	8	7	1		14	8	1		4	3	1	
4–10	68	58	0.87	(0.37–2.03)	101	55	1.02	(0.51–2.04)	61	51	1.74	(0.56–5.42)
> 10	41	35	1.02	(0.40–2.60)	68	37	1.54	(0.71–3.30)	54	45	2.48	(0.75–8.22)
MMSE												
Normal (24–30)	50	43	1		79	43	1		33	28	1	
Abnormal (< 24)	67	57	0.71	(0.43–1.19)	104	57	0.81	(0.53–1.25)	86	72	1.33	(0.76–2.33)
IADL												
No difficulties (8)	19	16	1		28	15	1		13	11	1	
Difficulties (0–7)	98	84	1.18	(0.58–2.41)	155	85	1.78	(0.99–3.22)	106	89	1.2	(0.53–2.70)
GDS-15												
Normal (0–6)	81	69	1		147	80	1		86	72	1	
Depressed (> 6)	36	31	**3.53**	**(1.99–6.23)**	36	20	1.68	(0.97–2.90)	33	28	**2.11**	**(1.15–3.87)**
EMS												
Normal (14–20)	80	68	1		142	78	1		68	67	1	
Abnormal (< 14)	37	32	**2.74**	**(1.48–5.09)**	41	22	1.41	(0.80–2.49)	51	43	**2.41**	**(1.28–4.54)**
TUG												
Normal (1–2)	33	28	1		70	38	1		36	30	1	
Abnormal (3–5)	78	67	1.44	(0.81–2.56)	102	56	0.8	(0.50–1.26)	69	58	0.75	(0.41–1.35)
Not known	6	5	1.55	(0.47–5.14)	11	6	1.08	(0.39–2.96)	14	12	1.23	(0.42–3.67)
Grip strength												
Normal (≥ 16 kg)	28	24	1		47	26	1		24	20	1	
Abnormal (< 16 kg)	56	48	0.77	(0.42–1.39)	98	54	0.89	(0.55–1.45)	82	69	1.2	(0.65–2.21)
Not known	33	28	0.78	(0.35–1.74)	38	21	0.66	(0.33–1.34)	13	11	0.6	(0.23–1.55)
MNA-SF												
Normal (12–14)	55	47	1		96	53	1		35	29	1	
Poor nutrition (< 12)	62	53	0.84	(0.50–1.41)	87	48	0.79	(0.51–1.22)	84	71	1.68	(0.96–2.92)
Fecal incontinence												
No	105	90	1		163	89	1		98	82	1	
Yes	12	10	2.31	(0.97–5.50)	20	11	**2.6**	**(1.22–5.54)**	21	18	**3.61**	**(1.65–7.90)**
Constipation												
No	45	39	1		61	33	1		38	32	1	
Yes	39	33	0.82	(0.47–1.44)	79	43	1.49	(0.95–2.35)	70	59	**1.78**	**(1.05–3.03)**
Not known	33	28	0.8	(0.38–1.67)	43	24	1.21	(0.63–2.31)	11	9	0.51	(0.20–1.28)
BMI												
23–28	55	47	1		79	43	1		41	35	1	
< 23	22	19	0.58	(0.31–1.05)	37	20	0.72	(0.44–1.19)	28	24	0.83	(0.45–1.53)
> 28	40	34	1.39	(0.82–2.34)	67	37	**1.8**	**(1.15–2.81)**	50	42	**2.81**	**(1.62–4.88)**
New falls after fracture												
No	81	69	1		121	66	1		80	67	1	
Yes	36	31	1.08	(0.64–1.82)	62	34	1.37	(0.89–2.11)	39	33	1.11	(0.66–1.87)

Results are shown by odds ratios (OR) with 95% Confidence intervals (CI). Statistically significant (*p* < 0.05) ORs are in bold

*ASA* American society of anesthesiologists-risk score, *MMSE* mini mental state examination, *IADL* instrumental activities of daily living, *GDS-15* geriatric depression scale, *EMS* elderly mobility scale, *TUG* timed up and go-test, *MNA-SF* mini nutritional assessment short form, *BMI* body mass index. Small numbers of missing values (*n* < 15) are not shown in table

### Statistical analyses

Groupwise comparisons between the continent and different UI subtype groups were performed using the Pearson’s Chi^2^ test or Fisher’s exact test. Multivariable-adjusted multinomial logistic regression analysis, where all the variables were simultaneously included in the model, with odds ratios (OR) and 95% confidence intervals (CI) was conducted to examine the associations of the outpatient domains with SUI, UUI and MUI at follow-up using the continent group as a reference. IBM SPSS Statistics version 27.0 for Windows software (SPSS Inc. Chicago, Illinois) was used for statistical analyses. All tests were two-sided and *p* values < 0.05 were considered statistically significant.

## Results

Of the original study population of 1,675 women, 638 (38%) were continent at the time of the fracture, 874 (52%) had UI, and data on continence status was missing in 163 (10%) of the cases. Mortality rate before the CGA was 18% (307 women). Of the 779 surviving patients attending the outpatient CGA, 360 (46%) were continent and 419 (54%) had UI 6 month post-fracture. Of the women with UI 117 (28%) had SUI, 183 (44%) had UUI, and 119 (28%) had MUI, respectively. Mean age of the patients was 82 ± 6, 91. Distributions of outpatient domains between the continent and different UI subtype groups are presented in Table 1. The continent women tended to be slightly younger, have fewer medications, less disability and impaired mobility, less depressive mood, and lower BMI than women suffering from any type of UI. Especially those affected by MUI tended to be older, in poorer physical condition according to the EMS, having more medications, malnutrition and FI compared to the other groups (Table 1).

In the multivariable model, depressed mood (OR 3.53, 95% CI 1.99–6.23) according to GDS-15 and impaired mobility and functional ability (OR 2.74, 95% CI 1.48–5.09) according to EMS were independently associated with SUI at 6 month post-fracture. Self-reported FI (OR 2.60, 95% CI 1.22–5.54) and BMI over 28 (OR 1.80, 95% CI 1.15–2.81) were independently associated with UUI. Depressive mood (OR 2.11, 95% CI 1.15–3.87), impaired mobility and functional ability (OR 2.41, 95% CI 1.28–4.54), FI (OR 3.61, 95% CI 1.65–7.90), BMI over 28 (OR 1.62, 95% CI 1.62–4.88) as well as self-reported constipation (OR 1.78, 95% CI 1.05–3.03) were all independently associated with MUI at 6 month post-fracture (Table 2).

## Discussion

As we have already established in our previous work [17] overall UI is very common among older female hip fracture patients with over half of the patients being affected in this study. This study sheds light on the different subtypes of UI in this patient group. UUI was the most common UI-type followed by MUI and SUI. The prevalences of different UI subtypes have varied greatly in previous studies due to differences in definitions, study populations, reporting, and age distributions of the participants. In studies including also older women, prevalences of SUI, UUI, and MUI have ranged between 14–26%, 6–50% and 14–31%, respectively [30–33]. The prevalence of UUI in our study lands on the higher end of the range compared to previous literature, reflecting our considerably old and selected multimorbid patient population.

Interestingly, in this study there was little difference in prevalences between MUI and SUI, although MUI has been established to dominate in older age groups [6, 8]. This might be partly explained by selection and survivor bias. Given our old and vulnerable study population, patients in poorest of health were more likely to die or drop out before the outpatient CGA and less likely to answer the questions on UI subtypes during the CGA, resulting in lower-than-expected prevalence of MUI. However, we found no studies directly examining prevalences of different UI subtypes among hip fracture patients. Hip fracture is a major trauma likely affecting the pelvic floor, and it is followed by a period of immobilization and rehabilitation when loss of muscle mass, including the pelvic floor, and function is to be expected. Since our study had a cross-sectional setting, we cannot establish causality, and this calls for further studies focusing on changes in the subtypes of UI in this patient population.

Depressive mood was associated with both SUI and MUI and the association wasn’t far from being significant with UUI. The association between depression and over-all UI has been demonstrated before in older women living in the community [34], and after a hip fracture [17]. Moreover, depression is a recognized risk factor for hip fractures [35], and patients recovering from a hip fracture are at risk of developing depression [36], suggesting a bidirectional relationship between the two conditions. The patients attended the outpatient CGA during the recovery period of the fracture when moving and toileting are expected to be slower and more burdensome because of pain and imbalance, possibly precipitating incontinence, and further declining patient’s quality of life and thus, negatively affecting mental wellbeing. Why the association was strongest for SUI calls for further studies with longitudinal design.

Impaired mobility and functional ability according to EMS were associated with SUI and MUI. We have previously established a bidirectional relationship between over-all UI and mobility in our study population: pre-fracture impaired mobility predicts incident UI [19], and pre-fracture UI predicts decline in mobility 1 year post-fracture [37]. Both TUG score and grip strength failed to reach statistical significance in our analysis, possibly due to relatively large proportion of missing data. Interestingly, UUI was not associated with abnormal EMS score. In a previous study by Fritel et al., an opposite result was found where UUI and not SUI was associated with limitations of motor and balance skills [38]. In another recent study, better lower extremity physical function protected from incident UUI and MUI [39]. The lack of association might be partly explained by our selected study population. The hip fracture and following surgery have major impact on patient’s mobility and possibly pelvic floor, which may have different impact depending on UI subtype. This calls for further studies with bigger samples**.**

Our analysis included data on new falls after the fracture. Even though falls tended to be more common among patients reporting any type of UI compared to their continent counterparts, new falls after the fracture were not associated with any UI type 6 month post-fracture in the multivariable-adjusted analysis. In previous literature, UI and especially UUI has been associated with an increased risk of falls [13, 14]. However, the underlying cause for this connection has been under some debate. Previously suggested simple rationale such as rushing to the toilet caused by the sense of urgency has been questioned [13, 40], and the current evidence suggests that likely both UI and falls are markers of underlying vulnerability and disability [14, 41, 42]. Our results concur with these findings, given that our analysis was adjusted with considerable number of factors (many of which represent disability and vulnerability) and our selected study population consisted of participants with high level of disability as already demonstrated in our previous works [17, 19]. Both UI and falls likely represent the vulnerable state of these patients without causal relationship.

BMI over 28 was associated with UUI and MUI in our study, the association being stronger for MUI. Obesity has been found to be a significant risk factor for any UI subtype in older women [43, 44]. Komesu et al. found age between 80 and 90 and BMI over 35 to be a predictors of incident MUI, as well as lower remission rates during a 2 year follow-up [18]. In another study by Pang et al., women aged 60 or over and with BMI over 24 had a higher predicted probability of remaining with or progressing to MUI from either SUI or UUI during a 4 year follow-up [45]. Since obesity also exerts challenges to mobilizing and rehabilitating the patient after a hip fracture, and on the other hand these patients frequently suffer from malnutrition [46] and thereby might carry the risk of sarcopenic obesity [47], preventing or managing UI by weight loss presents a clinical challenge in these patients.

FI was associated with both UUI and MUI 6 month post-fracture, again the association being stronger for MUI. Constipation was also independently associated only with MUI, but this result should be interpreted with caution, given that data on constipation was missing in nearly every third patient with SUI and every fifth with UUI. We demonstrated the association of constipation with over-all UI in our previous work [17]. Constipation is a known risk factor for UI with multidimensional causes such as insufficient mobility and hydration, as well as comorbidities [48, 49], all which patients with hip fracture are susceptible to. Coyne et al. demonstrated a significant overlap of UUI, constipation and FI in general population of both men and women aged 40 and over [50]. The association with FI might also be related to overuse of laxatives to treat constipation, or incidents of overflow FI related to severe constipation [50]. In a study by Botlero et al., loose FI was a risk factor for any type of UI independent of age or BMI in community-dwelling women [51]. UI and FI frequently coexist (i.e. double incontinence, DI) in our patient population with every tenth patient being affected 6 month post-fracture in our previous study. We demonstrated patients with DI to be an especially vulnerable group with higher disability and functional limitations compared to patients with UI only, and DI was strongly associated with pre-fracture UI [17]. We didn’t examine different subtypes of UI in our previous work, but in light of our new findings, it is likely that the subtype of UI which the patients with DI are affected by, is often either UUI or MUI.

The associated factors of both SUI and UUI converged in MUI, which is to be expected given the condition is defined as a combination of the other two [1]. Patients with MUI were the most vulnerable group in this study. In younger women evaluating UI subtype is important in selecting the appropriate treatment measures [3]. In our patients however, a CGA and rehabilitation plan is key in assessing UI, regardless of its subtype. Specific attention needs to be paid to physical function and mobility with an aim of regaining pre-fracture level. Of note, a multidimensional exercise treatment programme including pelvic floor muscle exercises has been proven to benefit older women not only with SUI but also with UUI and MUI [52]

According to best of our knowledge, this was one of the first studies aimed to examine factors associated with different UI subtypes among older female hip fracture patients. The strengths of our study were its real-world design, systematic data collection, and the comprehensive selection of well-known and standardized instruments with which the outpatient CGA was carried out. In addition, our results are representative of female hip fracture patients given that both patients living in assisted living accommodations or long-term care and having cognitive disorders were included in the study.

We acknowledge some limitations which should be considered when interpreting the results. First, incontinence symptoms were evaluated only with simple questions instead of validated questionnaires, and frequency, severity, time frame, or bother of the symptoms were not included in the data collection. Instead, several other significant assessments were included in the outpatient CGA. Second, comorbidities were not recorded in detail, and thus possible pre-existing urogynecological disorders were not known. Only ASA score represents the general health of the patient. However, ASA score has been established to correlate adequately with Charlson comorbidity index in hip fracture patients [53]. Third, our study concerned only women with hip fracture. We chose to concentrate on women because both hip fractures and continence problems are notably more common in women than in men and the pathophysiology between the sexes differs. Fourth, considering the relatively small sample sizes of the SUI and MUI groups, some caution is due when interpreting our results. Finally, the possibility of selection and survivor bias should be considered given that the data on different UI subtypes were available from less than half of the original study population. Nearly every fifth patient had died before the CGA, and those in poorest of health could not attend the CGA, thus true prevalences of especially UUI and MUI might be higher than presented in this study.

## Conclusions

All UI subtypes were associated with modifiable risk factors which should be taken into consideration in the management and rehabilitation of older women with hip fracture. Patients with MUI had most associated factors. In all, comprehensive geriatric assessment is key in assessing UI in older hip fracture patients, regardless of UI subtype.

## Data Availability

The datasets generated and analysed during the current study are not publicly available due to limitations of ethical approval involving the patient data and anonymity but are available from the corresponding author on reasonable request.
